# Protocol: Assessing the impact of interest‐holder engagement on guideline development: A systematic review

**DOI:** 10.1002/cl2.1444

**Published:** 2024-10-15

**Authors:** Lyubov Lytvyn, Jennifer Petkovic, Joanne Khabsa, Olivia Magwood, Pauline Campbell, Ian D. Graham, Kevin Pottie, Julia Bidonde, Heather Limburg, Danielle Pollock, Elie A. Akl, Thomas W. Concannon, Peter Tugwell

**Affiliations:** ^1^ Department of Health Research Methods, Evidence, and Impact McMaster University Hamilton Ontario Canada; ^2^ Bruyère Research Institute University of Ottawa Ottawa Ontario Canada; ^3^ American University of Beirut Medical Center, Clinical Research Institute Beirut Lebanon; ^4^ C.T. Lamont Primary Health Care Research Centre, Bruyere Research Institute Ottawa Ontario Canada; ^5^ Nursing, Midwifery and Allied Health Professions Research Unit Glasgow Caledonian University Glasgow UK; ^6^ School of Epidemiology and Public Health University of Ottawa Ottawa Ontario Canada; ^7^ Family Medicine Dalhousie University Halifax Nova Scotia Canada; ^8^ Norwegian Institute of Public Health Oslo Norway; ^9^ Public Health Agency of Canada Ottawa Ontario Canada; ^10^ University of Adelaide Adelaide South Australia Australia; ^11^ Department of Internal Medicine American University of Beirut Medical Center Beirut Lebanon; ^12^ The RAND Corporation Boston Massachusetts USA; ^13^ Clinical Epidemiology Program Ottawa Hospital Research Institute Ottawa Ontario Canada

**Keywords:** engagement, guidelines, impact, patient and public involvement, stakeholder

## Abstract

This is the protocol for a Campbell systematic review. The objectives are as follows. The objective of this review is to identify and synthesize empirical research on the impacts of interest‐holder engagement on the guideline development process and content. Our research questions are as follows: (1) What are the empirical examples of impact on the process in health guideline development across any of the 18 steps of the GIN‐McMaster checklist? (2) What are the empirical examples of impact on the content in health guideline development across any of the 18 steps of the GIN‐McMaster checklist?

## 1 BACKGROUND

Guidelines are an essential part of delivering safe and effective healthcare. High‐quality clinical practice guidelines are evidence‐based recommendations that are developed using a systematic approach to the evidence, weighing the balance of benefits and harms of policies and/or interventions (WHO Handbook for guideline development). Engaging relevant interest‐holders throughout the guideline development is considered to contribute to high‐quality guidelines (Brouwers et al., 2010; Neumann et al., 2016; Steinberg et al., 2011). Interest‐holders are defined as any individual or group who is responsible for or affected by health‐ and healthcare‐related decisions.

We previously used the term “stakeholders” to describe these groups of people. However, based on feedback from members of the MuSE Consortium, we have selected “interest‐holders” as our term moving forward. Interest‐holders may include patients, caregivers, members of the public, healthcare providers, principal investigators/researchers, peer review editors, health services payers, product makers, program managers, policymakers, and research funders (Concannon et al., 2012). Healthcare providers, patients, consumers, caregivers, and/or members of the public (or “laypersons”) are the interest‐holder groups that are most often involved in guideline development (Armstrong et al., 2017; Boivin et al., 2010; Cluzeau et al., 2012).

Interest‐holder input to both research and policy is supported by moral and ethical reasons. Interest‐holders have a democratic right to be involved in activity that may have an impact on them. Their engagement is also valuable for methodological reasons because it may improve the feasibility, acceptability, and uptake/implementation of guidelines. Interest‐holder engagement is increasingly required by funders (Arnstein, 1969; Cluzeau et al., 2012; Concannon et al., 2019; Greenhalgh et al., 2017; Minogue et al., 2018). Many guidelines, however, have limited or no engagement (Alonso‐Coello et al., 2010; Armstrong & Bloom, 2017; Gupta et al., 2015). Several reviews have highlighted a number of obstacles to engagement. These include lack of guidance on how to best to engage with interest‐holders and incorporate their input, limited opportunities for interest‐holders to contribute (e.g., insufficient outreach and support, power imbalances), inadequate resources (e.g., time, personnel, cost), and little understanding of the impact that interest‐holders have on the guideline process and final product (Boivin et al., 2018; Brouwers et al., 2010; Cluzeau et al., 2012; Greenhalgh et al., 2017).

### 1.1 Description of the condition

Guideline development generally follows a standard process, including topic selection and question formulation, evidence identification, evaluation, and synthesis, formulation of recommendations, and dissemination (chünemann et al., 2014). This process involves identifying and incorporating various perspectives and preferences, negotiation, and consensus building (Kunz et al., 2012). Guidelines should also include explicit consideration of other factors that may influence the recommendation, such as feasibility, acceptability, and resource considerations (WHO Handbook for guideline development; Kunz et al., 2012; Schünemann et al., 2014). The GIN‐McMaster Guideline Development Checklist includes 18 topics covering the guideline development process (Schünemann et al., 2014; Table 1).

**Table 1:** Draft framework categorizing impact outcomes.

	Examples of possible impact outcomes	
GIN‐McMaster Guideline Development Checklist Step	Impact on guideline process	Impact on guideline content
1. Organization, Budget, Planning and Training	Change/differences in cost/budget decisions Change/differences in timeline Change/differences in training provided	Not applicable
2. Priority Setting	Change/difference in priority setting methods	Change in guideline priorities
3. Guideline Group Membership	Change/difference in group selection methods	Not applicable
4. Establishing Guideline Group Processes	Change/difference in processes (e.g., modes or methods of meetings, voting, etc.)	Not applicable
5. Identifying Target Audience and Topic Selection	Change in methods for identifying topics	Change in guideline scope
6. Consumer and Stakeholder Involvement	Change in interest‐holder engagement policies/methods	Not applicable
7. Conflict of Interest Considerations	Change in conflict of interest policy or its application	Not applicable
8. (PICO) Question Generation	Differences in discussions about research question elements (population, intervention, comparator, patient/public/health system outcomes)	Change in any elements of the research question Differences in questions generated by guideline group with and without interest‐holders involved
9. Considering Importance of Outcomes and Interventions, Values, Preferences and Utilities	Disagreements about outcome priorities between interest‐holders involved in guideline and how they were resolved	Change in outcome priorities Differences in outcome priorities by guideline group with and without interest‐holders involved
10. Deciding What Evidence to Include and Searching for Evidence	Change in type of evidence considered eligible for guideline Change in search strategy (e.g., terms, databases)	Not applicable
11. Summarizing Evidence and Considering Additional Information	Differences in consideration of additional information	Change in information used to inform guideline
12. Judging Quality, Strength or Certainty of a Body of Evidence	Change/difference in methods for voting for quality, strength, certainty	Not applicable
13. Developing Recommendations and Determining Their Strength	Differences in EtD considerations	Change in recommendations Differences in recommendations generated by guideline group with and without interest‐holders involved Disagreements between interest‐holders involved in guideline and how they were resolved
14. Wording of Recommendations and of Considerations of Implementation, Feasibility and Equity	Not applicable	Change in wording of recommendations (e.g., phrasing) Change in wording of considerations of implementation, feasibility, and equity
15. Reporting and Peer Review	Differences in guideline content after peer review feedback from interest‐holders	Identification of gaps in reporting
16. Dissemination and Implementation	Not applicable	Change in dissemination strategies Change in implementation strategies
17. Evaluation and Use	Not applicable	Change in evaluation and use strategies (e.g., change in quality measures)
18. Updating	Change in the plan for updating (setting parameters for when an update should be considered, identifying the signal for updating)	Not applicable

Abbreviation: EtD, evidence to decision.

### 1.2 Description of the intervention and how it might work

Interest‐holder engagement refers to involving persons and/or organizations who may be affected by a health care or policy guideline in its development process (Cluzeau et al., 2012; Schünemann et al., 2014). Other terms commonly used to refer to engagement are involvement, collaboration, partnership, and co‐production. Engagement can occur at varying levels at any step of the guideline development process and may involve different methods, such as document review, focus group interviews, and guideline development group membership (Armstrong et al., 2017; Cluzeau et al., 2012; Del Campo et al., 2011). Notably, interest‐holder engagement is often not conceptualized as a discrete intervention, however, assessing the impact of interest‐holder engagement can be evaluated by treating “engagement” as the intervention, and examining the relationship between engagement and outcomes about guideline process and content.

**Figure 1:**
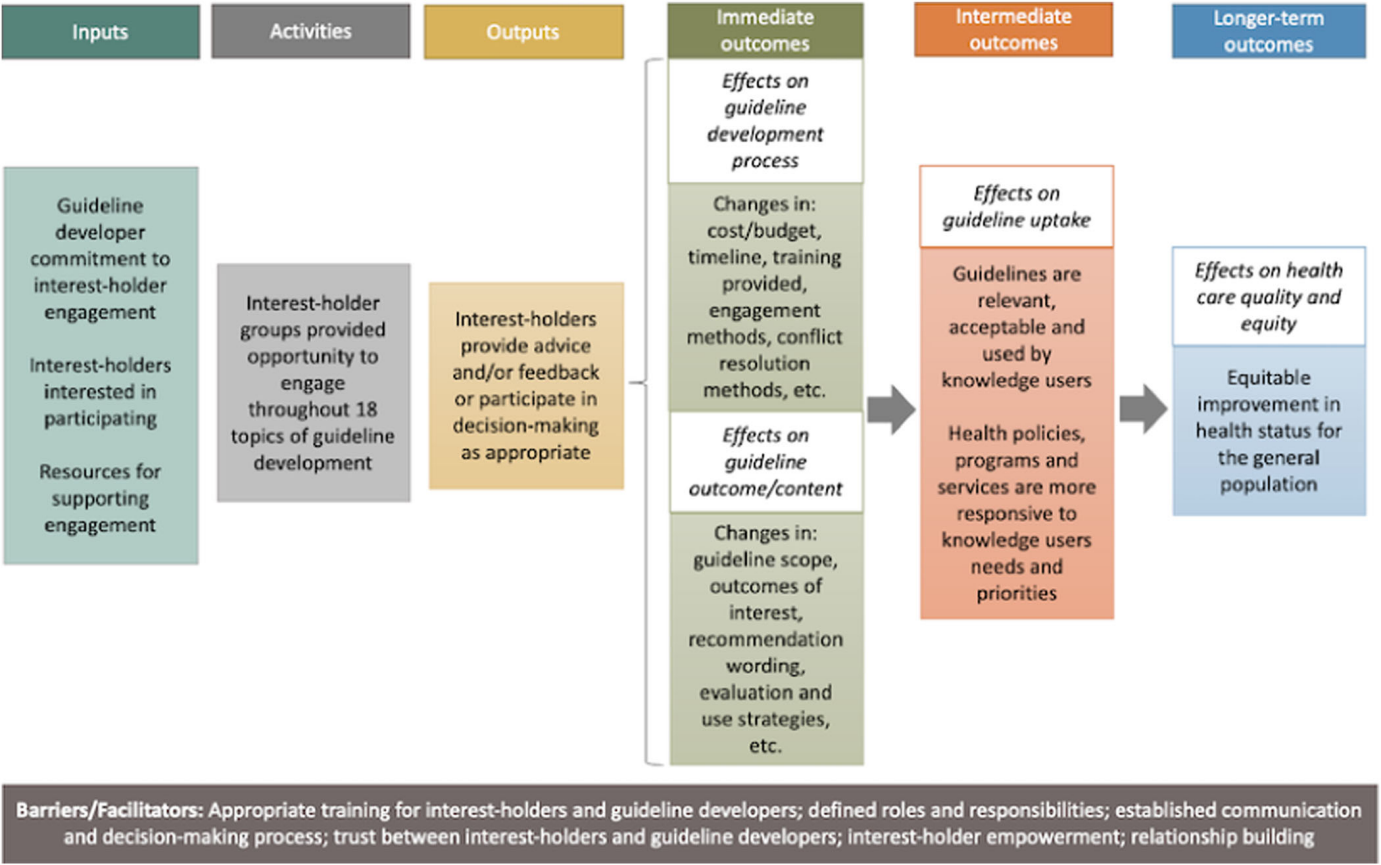
Logic model of interest‐holder engagement in guideline development.

We developed a logic model for the MuSE project to describe the activities, outputs, immediate outcomes, intermediate outcomes, and longer‐term outcomes, guiding our conceptualization of interest‐holder engagement in guideline development (Petkovic et al., 2020). We modified this logic model for this review to focus on the immediate outcomes and effects of engagement on guideline development process and guideline content (Figure 1).

Defining and measuring the impact of engagement in research and policy presents several challenges. Common issues are a lack of standardized engagement methods, limited conceptualization and planning, inadequate use of theory/frameworks, and poor reporting of both methods and impact outcomes (e.g., implicit or vague descriptions) (Boivin et al., 2010; Légaré et al., 2011; Staniszewska et al., 2011). One of the ways that a lack of standardization affects reporting is that engagement methods and outcomes (e.g., number of engaged interest‐holders) and impact (e.g., how those interest‐holders' engagement affected the guideline in some way) are used interchangeably. In a recent review of research partnerships by Mrklas et al., the authors found that explicit definitions for “outcomes” and “impact” were only available in less than half of the studies they identified, and there were inconsistencies in the definition and use of the terms (Mrklas et al., 2023). To improve clarity, we developed a draft conceptual framework to guide data extraction and categorization of outcomes, based on the 18 topics of the GIN‐McMaster Checklist, further described in “Methods” (Table 1). In this framework, the rows are the topics of guideline development, and for each topic, there are two columns, each to capture both the impact on the guideline development process and the impact on guideline content. As an example, we used this framework to demonstrate data extraction in three eligible studies (Table 2), with study‐level details about each impact outcome categorization (Table 3).

**Table 2:** Examples of eligible studies.

Study	Study design	Objective	Interest‐holder(s) engaged	Engagement methods (level of engagement)	Checklist topic(s)	Impact
Armstrong et al. (2018)	Cohort study	Compare the conduct and proposed research questions to be included in a guideline, from one guideline development group including patient representatives and one without	Patients and caregivers	Direct involvement in guideline development group (decision‐making)	(PICO) Question Generation	‐ Differences in discussions about research question elements (population, intervention, comparator, patient/public/health system outcomes) ‐ Change in elements of the research question ‐ Differences in questions generated
Campbell et al. (2017)	Case study	Determine the frequency and nature of changes made to draft guidance (*n* = 183 pieces) as a result of responses to public consultation	Providers, product‐makers, patients and caregivers	Public consultation (advice/feedback)	Summarizing Evidence and Considering Additional Information	‐ Change in information used to inform guideline
					Developing Recommendations and Determining their Strength	‐ Change in recommendations
					Wording of Recommendations and of Considerations of Implementation, Feasibility and Equity	‐ Change in wording of recommendations (e.g., phrasing)
Fraenkel et al. (2016)	Cohort study	Compare strength and direction of recommendations developed by a physician‐dominated voting panel and a patients‐only panel	Patients	Direct involvement in guideline development group (decision‐making)	Developing Recommendations and Determining their Strength	‐ Differences in recommendations generated ‐ Differences in EtD factor considerations^a^

Abbreviations: EtD, evidence to decision; PICO, Population, Intervention, Comparator, Outcomes.

a The patient voting panel mentioned that evidence tables did not include patient‐important outcomes which they argued should be considered when weighing benefits versus harms. However, given that the scope of the study did not include changing the research question or evidence, this was categorized as differences in EtD factor considerations rather than differences in discussions about research question.

**Table 3:** Impact outcomes – Study level details.

Study	Impact outcome	Details
Armstrong et al. (2018)	Differences in discussions about research question elements (population, intervention, comparator, patient/public/health system outcomes)	[The] experimental group spent more time discussing reasons it is important for patients and families to have a diagnosis in the setting of cognitive impairment. The patient representatives emphasized the importance of having a diagnosis in general, getting closure, validation that something is wrong, reduction in uncertainty, avoidance of unnecessary testing, understanding prognosis, linking to services and support, being able to plan one's life, enabling formal advance planning, accessing disability and employment protections, accessing targeted treatment and care, and gaining increased control.
	Change in elements of the research question	Proposed guideline questions, benefits, and harms were largely similar between groups, but only the experimental group proposed outcomes relating to development of cognitive impairment at certain time points (rather than considering development of dementia as a binary outcome) and proposed rate of progression as an outcome.
	Differences in questions generated	The experimental group drafted eight over‐arching PICOT questions versus four drafted by the control group, but the control group nested multiple populations within each PICOT question.
Campbell et al. (2017)	Change in information used to inform guideline	In 22.4% (41/183) of public consultations, responses were received which proffered additional empirical studies that were subsequently added to the procedure overview document, which is used by the committee as a basis for its deliberations. These changes were in addition to those made to the draft guidance, listed above. Typically, the additional studies were ones published after NICE's original literature review, which had also been retrieved by a routine updated search, but occasionally undiscovered studies were identified. The additional studies were considered by the committee, alongside the evidence already reviewed, to decide whether their findings should change any aspect of the draft guidance.
	Change in recommendations	Responses were received during 159/183 (86.9%) periods of public consultation, from a total of 853 people or organizations (median number per consultation 3; range 0–82; interquartile range 1–5). Changes were made to draft guidance following 136 (74.3%) consultations. These changes were to the category (2.7%) or wording (8.7%) of the main recommendation; to other [sections of the] recommendations (about consent, patient selection, training and future research) (31.1%); and to other sections of guidance [apart from the recommendations] (description of the procedure and of the evidence on its efficacy and safety) (70.5%).
	Change in wording of recommendations (e.g., phrasing)	
Fraenkel et al. (2016)	Differences in recommendations generated	The patient panel was able to develop recommendations for 16 of the 18 PICO questions with [moderate to high certainty] level of evidence. They chose not to vote on 2 questions (Questions 10 and 13) because they thought that they did not have enough direct data to support a recommendation. For 13 of the remaining 16 questions, the patient panel voted in the same direction as the physician‐dominated panel. The strength of the recommendation was the same across both panels for 10 of these 13 recommendations.
	Differences in EtD factor considerations	[The patients] noted that the evidence tables did not include some of the adverse events (such as gastrointestinal side effects, light‐headedness, and general malaise), which they argued should be considered when weighing benefits versus harms. The patients stated that the data included in the evidence tables were not always sufficiently detailed in order for them to accurately gauge harm. The patient panel also thought that quality of life (reflecting domains besides physical function) should be included as an outcome in the evidence tables.

Abbreviations: EtD, evidence to decision; NICE, National Institute for Health and Care Excellence; PICOT, Population, Intervention, Comparator, Outcome, Timeframe.

Impact is frequently reported as a binary outcome, categorized as either “positive” or “negative” impact. Often when impact is reported, it is synonymous with positive impact or “benefit.” The framing of impact may be related to how authors want their interest‐holder engagement to be perceived. It is usually described as a beneficial experience for both the authors and the interest‐holders involved, for example, for ethical, financial, and process‐related reasons, or to justify time and resources spent by those involved. Negative impact is less frequently reported; and when it is described, it is downplayed with study authors focusing on the process‐related challenges that directly affect them (e.g., cost and resources required). The negative impact on other interest‐holders is often overlooked (Hoekstra et al., 2020; Mrklas et al., 2022). To ensure we capture all relevant outcomes, we use the term “impact” regardless of the direction of effect.

### 1.3 Why it is important to do this review

There are currently no up‐to‐date reviews focused on the impact of interest‐holder engagement in guideline development. To inform guideline developers about how to engage interest‐holders more meaningfully, the impact of different approaches to engagement needs to be summarized and compared.

Meaningful engagement requires significant investment, both financial and in terms of time. Evaluating the impact of interest‐holder engagement in research and policy is important for demonstrating the value (i.e., return on investment) and improving engagement practices (e.g., by identifying effects of different approaches) (Hoekstra et al., 2020; Vat et al., 2021). The value of interest‐holder engagement in research and policy has been widely reported in several reviews (Brett et al., 2014a, 2014b; Crocker et al., 2017; Forsythe et al., 2018; Mockford et al., 2012; Staniszewska et al., 2011; van de Bovenkamp & Zuiderent‐Jerak, 2015). Positive contributions include shaping and prioritizing research questions so that they are more relevant to end users, choosing outcomes that are most important to patients, enabling generalizability and a more comprehensive interpretation of results, enhancing mutual learning, as well as improved communication, dissemination, acceptability, and uptake of findings (Brett et al., 2014a, 2014b; Crocker et al., 2017; Forsythe et al., 2018; Mockford et al., 2012; Staniszewska et al., 2011). Impact needs to be contextualized, as it is affected by the engagement process, such as which interest‐holders were involved and at which stage(s) of the guideline development process. Other factors that may affect impact are the type of engagement strategies used, how much training/support was received, and the level of engagement (advice/feedback or decision‐making) (Brett et al., 2014b; Crocker et al., 2017). Impact could also be affected by the guideline topic, and the relationships between different interest‐holders (Brett et al., 2014b; Crocker et al., 2017). An additional consideration is that some authors report that the more engagement there was with interest‐holders (e.g., engaging early and often), the more challenging it was to identify the specific impacts interest‐holders may have had (Crocker et al., 2017). There are several tools that help facilitate planning, evaluation, and reporting of interest‐holder engagement methods and impact, though their focus is on patients and the public as interest‐holders and these tools have limited application in guideline development (Table 4).

**Table 4:** Current tools to facilitate planning, evaluation, and reporting of methods and impact of interest‐holder engagement in research.

Tool		
Guidance for Reporting Involvement of Patients and Public (GRIPP2, and GRIPP2‐Short Form) (Staniszewska et al., 2017)	Brief description	*Long form reporting guidance:* eight sections (abstract, background, aims, methods, capture or measurement of PPI impact, economic assessment, study results, and discussion and conclusions), each with 1–9 items (e.g., provide a clear description of methods by which patients and the public were involved, report any conceptual or theoretical development in PPI that emerged). *Short form reporting guidance:* five sections (abstract, methods, results, discussion and conclusions, reflections/critical perspective), each with one item (e.g., report the aim of PPI in the study; comment critically on the study, reflecting on the things that went well and those that did not, so others can learn from this experience).
	Method of development	Systematic review of 55+ studies, Delphi study, and face‐to‐face consensus meeting.
	Application in practice guidelines	Not known to be used in this context. Used in primary research and evidence syntheses.
Patient Engagement In Research Scale (PEIRS‐22) (Hamilton et al., 2018, 2021)	Brief description	Self‐administered questionnaire completed by patient partners or family caregiver partners to assess the degree of meaningful engagement in research.
	Method of development	Questionnaire items identified through in‐depth interviews and published literature, e‐Delphi survey to refine and select items, and cognitive interviewing participants understanding and opinions of each item and the structure of the scale. Validation study resulted in shortened scale from 37 to 22 items.
	Application in practice guidelines	Not known to be used in this context. Used in primary research.
Public and Patient Engagement Evaluation Tool (PPEET) (Boivin et al., 2018; Vat et al., 2021)	Brief description	*Survey:* Three modules (planning, assessing engagement, assessing impact) including questions on a 5‐point Likert scale, ranging from strongly disagree to strongly agree. The questions are related to the planning and integrity of engagement methods (e.g., were financial, logistical and information needs of participants were accommodated; did engagement component added value to the project it supported), and the impact of engagement (e.g., was output generated from the engagement component associated with this project was considered by those in a position to act on it). The impact module also includes a free‐text table to describe each stakeholder influence and its amount (none, small amount, moderate amount, a lot, not sure). All modules also have free‐text questions to add detail to the rating scales.
	Method of development	Systematic review of 27 evaluation tools, and stakeholder involvement in iterative development.
	Application in guideline development	Not generally used in guideline development context. Recently published validation study on using a long versus short version to evaluate engagement in guideline development (Moore et al., DOI: 10.1016/j.jclinepi.2021.11.034). Used in primary research, particularly health services research.
Taxonomy of impact measures of community stakeholder engagement in clinical and translational research (Stallings et al., 2019)	Brief description	*List of measures/metrics:* Metrics are grouped into two main categories, outcome metrics (three domains: internal, external, and aggregate outcomes) and process metrics (four domains: direct process metrics, surrogate process metrics, aggregate process metrics, and preconditions for engagement). Examples included participant metrics such as knowledge and satisfaction (outcome metrics, internal), involvement since first stage of decision process (process metrics, direct), and attendance (process metrics, surrogate).
	Method of development	Systematic review of 23 evaluation tools.
	Application in guideline development	Not used in guideline development context. Used in primary research, particularly health services research.
Taxonomy of metrics of patient engagement in healthcare organization and system‐level decision‐making (Dukhanin et al., 2018)	Brief description	*List of measures/metrics:* Research is categorized into seven stages (pre‐research, infrastructure, study design, implementation, analysis, dissemination, and post‐research), and for each stage there are activity clusters (e.g., proposal development, data analysis). For each cluster there are conceptual statements about activities (e.g., identify issues of greatest importance to community stakeholders, provide alternative interpretation of research results), and examples of associated metrics (e.g., number of ideas generated by community stakeholders, presence of stakeholder authors on manuscript).
	Method of development	Stakeholder‐identified items, validation, and refinement.
	Application in guideline development	Not used in guideline development context. Used in primary research.
Review of reviews on principles, strategies, outcomes and impacts of research partnership approaches (Hoekstra et al., 2020)	Brief description	*List of measures/metrics:* Authors identified 82 outcomes/impacts, synthesized them into 20 overarching outcomes/impacts, and clustered them into five subcategories: (1) outcomes and impacts on researchers conducting the partnership research (individual‐level); (2) outcomes and impacts on the stakeholder(s) (individual‐level); (3) outcomes and impacts on the relationship between researchers and stakeholders (partnership‐level); (4) outcomes and impacts on the broader community or society; and (5) outcomes and impacts on the research process.
	Method of development	Review of 74 reviews of research partnerships.
	Application in guideline development	Not used in guideline development context. Used in primary research and evidence syntheses.

There is a need for comprehensive, evidence‐based international standard guidance for interest‐holder engagement in guideline development (Petkovic et al., 2020). The MuSE Consortium was established in 2015 and includes over 140 individuals and groups internationally with a shared interest in developing methods and approaches for involving interest‐holders in health research and policy (Petkovic et al., 2020). This guidance for interest‐holder engagement in guidelines will be an extension of the GIN‐McMaster Guideline Development Checklist (Schünemann et al., 2014) and will be informed by a series of four reviews, summarizing evidence on: (1) guidance for interest‐holder engagement in guideline development (Petkovic et al., 2022), (2) barriers and facilitators to interest‐holder engagement in guideline development (Magwood et al., 2022), (3) managing conflicts of interest in interest‐holder engagement in guideline development (Khabsa et al., 2022), and (4) measuring the impact of interest‐holder engagement in guideline development (this review). Members of the MuSE consortium, including all our identified interest‐holder groups, have been involved in the planning of these reviews.

This review will synthesize existing empirical evidence on the impact of interest‐holder engagement on guideline development. We will focus on specific examples where authors intended to report a direct relationship between interest‐holders' contributions, and how this affected the guideline process or content across the 18 topics of the GIN‐McMaster Checklist (Schünemann et al., 2014). Impact on interest‐holders or guideline developers themselves (e.g., satisfaction, learning) and the general practice of engagement (e.g., trust, power dynamics, relationship building), while valuable, are out of the scope of this review. However, these issues will be explored in our review on barriers and facilitators to interest‐holder engagement in guideline development (Magwood et al., 2022).

The findings of this review, along with the other three in the series, may assist organizations who develop healthcare, public health, and health policy guidelines, to identify effective ways to involve multiple interest‐holders in the guideline development process to promote the development of relevant, high‐quality, and transparent guidelines.

#### 1.3.1 Summary of existing reviews

To the best of our knowledge, there are no reviews of the impact of interest‐holder engagement in guideline development. However, there are several syntheses focused on research and policy. A systematic review by Brett et al. identified 66 studies published between 1995 and 2012 that reported the impact of patient and public involvement on health and social care research (Brett et al., 2014b). Some of the positive impacts reported were the development of more relevant research objectives, research questions, questionnaires and interview schedules, and recruitment strategies, as well as enhanced implementation and dissemination of study results. Some negative impacts were also reported, such as the increased time and cost required. A linked review by Mockford et al. identified 28 studies published between 1997 and 2009 that report the impact of patient and public involvement on UK National Health Service healthcare services and costs (Mockford et al., 2012). They identified examples of impacts of engagement on healthcare services related to service planning and development, information development and dissemination, and changing attitudes of service users and providers. Reporting of cost was limited and only involved costs associated with particular involvement activities. Identified issues included poor conceptualization/theoretical underpinning of involvement, and that measurements were not validated. These two reviews were the evidence base that informed the development of the Guidance for Reporting Involvement of Patients and the Public (GRIPP2 and GRIPP2‐short form [Staniszewska et al., 2017]) reporting guidance, described in Table 4.

Another review of patient engagement evaluation tools assessed 27 tools, published between 1980 and 2016, on scientific rigor, inclusion of patient and public perspective, comprehensiveness, and usability (Boivin et al., 2018). Most of the tools identified were designed to evaluate key dimensions of patient and public engagement, such as process, context, or perceived self‐reported impact. However, the authors found that only a small number of instruments are informed by a comprehensive literature review, resulting in duplication of tools and misalignment with the most important dimensions of engagement. In addition, authors reported that tools lacked explicit conceptual frameworks, which limited the results of the empirical evaluation. The authors also highlighted that interest‐holders were infrequently involved in the tool development process and that the usability of the tools required high research‐related literacy. The Public and Patient Engagement Evaluation Tool (PPEET) was developed and informed by this review (Boivin et al., 2018; Vat et al., 2021) (Table 4).

Our review will build on the previous findings from these linked reviews by focusing specifically on guidelines, addressing a clear gap in the evidence base. Our review will expand on these related reviews in three key areas: first, we will focus specifically on guideline development, and use a broad search. Second, our review will adopt a broader definition of interest‐holder and will examine the evidence of impact by interest‐holder category, where possible. Third, we will examine the impact of interest‐holder engagement on the guideline development process as outlined by the GIN‐McMaster Guideline Development Checklist.

## 2 OBJECTIVES

The objective of this review is to identify and synthesize empirical research on the impacts of interest‐holder engagement on the guideline development process and content. Our research questions are as follows:

1. What are the empirical examples of impact on the process in health guideline development across any of the 18 steps of the GIN‐McMaster checklist?
2. What are the empirical examples of impact on the content in health guideline development across any of the 18 steps of the GIN‐McMaster checklist?

## 3 METHODS

### 3.1 Criteria for considering studies for this review

#### 3.1.1 Types of studies

This review will include quantitative, qualitative and mixed‐methods primary studies. This includes case studies, mixed‐methods studies, randomized trials, non‐randomized studies (e.g., cohort studies, before and after studies, cross‐sectional studies), process evaluation studies, policy analysis studies, and qualitative studies. Narrative reviews, conference abstracts, commentaries, editorials, and protocols will be excluded. Systematic reviews will be eligible, but only for reference checking.

#### 3.1.2 Types of participants

The population is interest‐holders in guideline development. We define interest‐holders as “any individual or group who is responsible for or affected by health‐ and healthcare‐related decisions” (Concannon et al., 2012).

The following interest‐holder groups will be included:

1. Patients, families, and caregivers, for example, individual patients, their caregivers, families, and patient and consumer advocacy organizations.
2. Public, individual persons, who may or may not be part of advocacy organizations.
3. Providers of healthcare, for example, nurses, physicians, pharmacists, mental health counselors, community‐based workers.
4. Payers/Purchasers of health services, for example, insurers, individuals with deductibles, others responsible, employers, governments and other entities responsible for underwriting the cost of care or for reimbursement for health‐related interventions.
5. Payers/funders of health research, for example, research councils, charities, government departments, international organizations.
6. Policymakers, for example, policy‐making entities such as governments and professional associations.
7. Product‐makers, for example, drug/device manufacturers.
8. Principal investigators, for example, researchers and all members of the research team.
9. Program managers (e.g., managers/directors/administrators) and individuals who plan, lead, oversee, or deliver any program that provides public health, community services, or clinical care (e.g., budgeting, hiring, staffing, organizing, coordinating, reporting).
10. Peer review editors (e.g., individuals who manage peer review processes or edit peer‐reviewed research).

#### 3.1.3 Types of interventions

Eligible studies will describe interest‐holder engagement in the development of clinical, public health, and health system guidelines. Example methods for engagement include eliciting comments via document review, conducting focus group interviews about specific issues, and having interest‐holders as guideline development group core members throughout all steps of the development process (Armstrong et al., 2017; Cluzeau et al., 2012; Del Campo et al., 2011). We will use the GIN‐McMaster Guideline Development Checklist to categorize interest‐holder engagement throughout different aspects of guideline development. Within each GIN‐McMaster Checklist topic, we will define the level of engagement as either providing advice/feedback or participating in decision‐making, which was adapted from previous work (Crowe, 2017; Oliver et al., 2008; Pollock et al., 2019).

### 3.2 Outcome measures

#### 3.2.1 Critical outcomes

The primary outcome is empirical evidence of the impact of interest‐holder engagement on guideline development. To guide data extraction of outcomes related to impact, a draft conceptual framework was developed. The framework was based on co‐author suggestions of impact related to the 18 topics and 146 items of the GIN‐McMaster checklist, previously published research on evaluation and reporting of impact of interest‐holder engagement in health research (Table 4), and pilot data extraction from eligible studies for the review (Table 1). As an example, we extracted data from three eligible studies (Armstrong et al., 2018; Campbell et al., 2017; Fraenkel et al., 2016; Table 2), and specified study‐level details about impact outcomes (Table 3). We will report both conceptual statements about authors' perceptions of impact, captured in qualitative outcomes (e.g., statements about whether interest‐holders had an impact on the prioritization of research questions), as well as specific impact metrics, captured in quantitative outcomes (e.g., X number of research questions were identified/modified based on interest‐holder input). For example, if authors report that engaging interest‐holders had an impact on which patient‐important outcomes were included in the guideline(s), this will be reported as an impact even if the specific information about how the impact was conceptualized was not adequately described (e.g., no description of how many outcomes were identified/modified).

It is important to note that this review is focused on empirical data published about impact on the guideline development process according to the GIN‐McMaster Guideline Development Checklist topics. We are focused on the outcomes related to the impact of interest‐holder engagement on the guideline process or content. A limitation of our review is that we may not adequately capture guidelines that have been co‐produced with interest‐holders as these will be unlikely to isolate specific contributions according to interest‐holder group (Masterson et al., 2022).

#### 3.2.2 Important outcomes

The outcomes that we will extract in this review were not prioritized based on importance and have equal consideration.

### 3.3 Search methods for identification of studies

This review is part of a series of four reviews conducted by the MuSE working group on interest‐holder engagement in guideline development. A single sensitive search strategy for all four reviews was developed in consultation with medical librarians.

#### 3.3.1 Electronic searches

The following databases will be searched: MEDLINE (OVID), CINAHL (EBSCO), EMBASE (OVID), PsycINFO (OVID), AMED (OVID), SCOPUS, and Sociological Abstracts (see Supporting Information).

#### 3.3.2 Searching other resources

To identify gray literature, we will conduct the following supplementary searches:

- Websites of agencies who actively engage interest‐holder groups. This will include, but is not limited to the Agency for Healthcare Research and Quality (AHRQ), Canadian Institutes of Health Research (CIHR) Strategy for Patient‐Oriented Research (SPOR), the National Institute of Health Research (NIHR) INVOLVE project, Guidelines International Network patient and public involvement group (GIN‐PUBLIC), the National Institute for Health and Care Excellence (NICE), and the Patient‐Centered Outcomes Research Institute (PCORI).
- Websites of guideline‐producing agencies, such as the National Institute for Health and Care Excellence (NICE), Australia's National Health Medical Research Council (NHMRC), Canadian Task Force on Preventative Health Care (CTFPHC), United States Preventative Services Taskforce (USPSTF), Scottish Intercollegiate Guidelines Network (SIGN), and the WHO.
- Contacting experts and wider networks for relevant studies: we will invite members of the MuSE Consortium to suggest gray literature sources, and we plan to broaden the search by soliciting suggestions via social media, such as Twitter.

##### Setting

No restrictions on type of guideline.

##### Language

No restrictions on language will be applied.

##### Publication date

No restrictions on publication date will be applied.

##### Citations and reference lists

We will review reference lists of relevant reviews to identify eligible primary studies for inclusion. We will do forward and backward searching of included studies, using the *citationchaser* tool (Haddaway et al., 2022).

### 3.4 Data collection and analysis

#### 3.4.1 Selection of studies

A two‐part study selection process will be used: (1) a title and abstract review and (2) full‐text review. Studies will be imported into Covidence and de‐duplicated. Pairs of review authors will independently assess all potential studies and documents using a priori inclusion and exclusion criteria. We will resolve any disagreements through discussion or, if required, we will consult a third review author.

We anticipate a variety of study designs, including comparative cohorts and narrative descriptions of case examples of engagement practices and their effects.

#### 3.4.2 Data extraction and management

We will extract the variables below. Data extraction will be done by two reviewers independently.

Study characteristics:

- Study setting (e.g., country/countries for which the guideline is being developed, guideline development body/organization).
- Characteristics of the guideline development panel.
- Guideline topic(s).
- Study design.
- Study objective(s).
- Number of guidelines addressed.
- Timeframe of guidelines addressed.
- Approach to guideline development used (e.g., GRADE).
- Funding.
- Budgeting/financial cost of engaging interest‐holders.

Interest‐holder engagement methodology:

- Theoretical model/framework used to guide interest‐holder engagement.
- Interest‐holder characteristics, sampling, and recruitment.
- Types of interest‐holders engaged.
- Number of interest‐holders engaged.
- Engagement methods.
- Training/support provided to interest‐holders.
- Frequency of engagement.
- Step of guideline development in which interest‐holders were engaged.
- Level(s) of engagement (i.e., advice/feedback or decision‐making).

Methods of impact assessment:

- Definition of impact.
- Data collection.
- Data analysis.
- Impact evaluator (e.g., who is reporting impact outcome[s]).
  Outcomes:
- Impact of interest‐holder engagement (positive and negative), on process or content, mapped to the GIN‐McMaster Guideline Development Checklist.

##### Multiple documents relating to the same study

If findings are reported in multiple documents, we will review all of the documents. We will select data from the study that we believe to be the most recent. Where additional unique, relevant outcome assessments are reported in other documents (e.g., secondary analysis reports), we will also make use of those documents. All documents relating to a single study will be listed in the included studies table.

#### 3.4.3 Risk of bias assessment in included studies

We anticipate that our included studies will have varied study designs, including comparative cohort studies and case studies. For quality appraisal of all included studies, we will use the Quality Assessment with Diverse Studies (QuADS) tool (Harrison et al., 2021). This tool was developed to determine the methodological and reporting quality of mixed‐ and multi‐method studies in systematic reviews of health services research and found to have substantial inter‐rater reliability (*k* = 0.66), as well as face and content validity for such applications. Using a single appraisal tool will facilitate reporting and comparison of quality across all studies included in the review, as opposed to using different tools depending on study design.

Critical appraisal will be done independently and in duplicate. Disagreements will be resolved by consensus, and, if needed, a third party will be consulted.

#### 3.4.4 Measures of treatment effect

Reviewers will extract all relevant data from each study following our draft conceptual framework (Table 1) using Google Sheets, a web‐based spreadsheet application (Google LLC, 2024). For quantitative outcomes, we will extract data on summary statistics (e.g., proportion of recommendations changed with interest‐holder input). For qualitative outcomes, we will extract all relevant data reported by study authors, including participant quotations from interviews or focus groups, and descriptive statements.

Findings will be organized by GIN‐McMaster checklist topic (e.g., Topic 13 – Developing recommendations and determining their strength), whether the impact is on guideline process or guideline content, and the category of the type of impact (e.g., change in wording of recommendations). The findings will be further classified by which interest‐holders are engaged, and at which level of engagement (i.e., advice/feedback or decision‐making). We will extract both positive or negative impacts. The framework may be iteratively adjusted based on empirical data from studies.

Data will be extracted independently and in duplicate. Discrepancies in the data extraction process will be resolved by consensus, and a third reviewer will be consulted if needed.

#### 3.4.5 Unit of analysis issues

We do not anticipate unit of analysis issues.

#### 3.4.6 Dealing with missing data

We will contact authors of included studies for further information, if necessary.

#### 3.4.7 Reporting bias assessment

We do not plan to assess reporting bias, however, we will discuss which are the guideline development topics where impact is seldom evaluated and reported.

#### 3.4.8 Synthesis methods

If there are two or more studies that report the same quantitative impact outcome measure, we will conduct a meta‐analysis following established methods available from the Cochrane Handbook (Deeks et al., 2023). However, given the expected heterogeneity in study designs, meta‐analysis may not be appropriate or possible, in which case we will conduct a synthesis without meta‐analysis (Campbell et al., 2020). For qualitative impact outcome measures, the choice of synthesis method will be determined after the evidence is known, however, it is likely that we will use thematic synthesis, guided by our conceptual framework of impact (Flemming et al., 2019).

Quantitative and qualitative data will be integrated in a tabular form. Our findings will be organized according to the 18 topics of the GIN‐McMaster Guideline Development Checklist, and the 10 interest‐holder categories.

We do not plan to systematically assess heterogeneity, however, we will examine variations across the included studies. Differences in study characteristics (e.g., study setting), engagement methodology (e.g., type of interest‐holders included, step of guideline development in which interest‐holders were engaged, level(s) of engagement), and methods of impact assessment (e.g., definition of impact, impact evaluator) may result in differences in findings across studies.

#### 3.4.9 Investigation of heterogeneity and subgroup analysis

We do not plan to conduct subgroup analyses.

##### Equity‐related assessment

Desirable interest‐holder engagement should include the equitable inclusion of different interest‐holder groups, particularly those who are known to be typically underrepresented. We use the term underrepresented in this context to refer to individuals or groups where (1) they are typically excluded from guideline development and implementation, and (2) they may experience health inequities. We will capture relevant population characteristics using the PROGRESS‐Plus framework, including place of residence, race/ethnicity/culture/language, occupation, gender/sex, religion, education, socioeconomic status, and social capital and other characteristics, such as disability and sexual orientation (O'Neill et al., 2014).

#### 3.4.10 Sensitivity analysis

We do not plan to conduct sensitivity analyses.

#### 3.4.11 Certainty of the evidence assessment

If there will be a synthesis of two or more quantitative outcomes, we will assess the certainty of the body of evidence using the Grading of Recommendations Assessment, Development and Evaluation (GRADE) approach (Guyatt et al., 2008). For qualitative findings, if there is sufficient data available we will assess the certainty using the GRADE‐Confidence in the Evidence from Reviews of Qualitative Research (CerQUAL) approach (Lewin et al., 2018).

We will report this review following the Preferred Reporting of Systematic Reviews and Meta‐Analysis (PRISMA) reporting, the Synthesis Without Meta‐analysis (SWiM) reporting guideline, and the Enhancing transparency in reporting the synthesis of qualitative research (ENTREQ) reporting guideline, as appropriate (Campbell et al., 2020; Page et al., 2021; Tong et al., 2012).

### 3.5 Consumer involvement

No consumers were included in this systematic review, however, consumers are included in the MuSE project as co‐leads of the patient and the public interest‐holder categories, as well as participants in a survey that asked interest‐holders to categorize whether they wanted to be in a decision‐making or advice/feedback role across the GIN‐McMaster guideline development checklist and one‐on‐one interview participants that expanded on the questions of the survey (Petkovic et al., 2020).

## CONTRIBUTIONS OF AUTHORS

Conceiving the review: PT, TWC, JP

Designing the review: JP, LL, OM, PT, TWC

Coordinating the review: LL, JP

Writing the protocol: LL, JP, JK, OM

Providing general advice on the review: DP, JB, PC, PT, TWC, IDG, HL, EAA, KP Securing funding for the review: PT, JP.

## DECLARATIONS OF INTEREST

VW is editor‐in‐chief of the Campbell Collaboration. This review will be handled by an independent editor, and the co‐chairs of the relevant group will act in lieu of editor‐in‐chief. TC has developed and published several peer‐reviewed publications that could potentially be included in the review. TC currently holds one research contract with the Patient‐Centred Outcomes Research Institute and another with the Pharmaceutical Research and Manufacturers of America Foundation that address a similar topic.

## SOURCES OF SUPPORT

### Internal sources

- There were no other sources of support for this review.

### External sources

- Canadian Institutes of Health Research, Canada Project Grant.

## REGISTRATION AND PROTOCOL

This protocol is registered in Campbell Systematic Reviews.

## DATA, CODE, AND OTHER MATERIALS

All data extracted during this systematic review will be available to the public upon request. No specific code or other materials are applicable for this evidence synthesis.

## References

[cl21444-bib-0001] Alonso‐Coello, P. , Irfan, A. , Sola, I. , Gich, I. , Delgado‐Noguera, M. , Rigau, D. , Tort, S. , Bonfill, X. , Burgers, J. , & Schunemann, H. (2010). The quality of clinical practice guidelines over the last two decades: A systematic review of guideline appraisal studies. BMJ Quality & Safety, 19(6), e58. 10.1136/qshc.2010.042077 21127089

[cl21444-bib-0002] Armstrong, M. J. , & Bloom, J. A. (2017). Patient involvement in guidelines is poor five years after institute of medicine standards: Review of guideline methodologies. Research Involvement and Engagement, 3(1), 19. 29062544 10.1186/s40900-017-0070-2 PMC5623959

[cl21444-bib-0003] Armstrong, M. J. , Mullins, C. D. , Gronseth, G. S. , & Gagliardi, A. R. (2018). Impact of patient involvement on clinical practice guideline development: A parallel group study. Implementation Science, 13, 55. 29661195 10.1186/s13012-018-0745-6 PMC5902835

[cl21444-bib-0004] Armstrong, M. J. , Rueda, J. D. , Gronseth, G. S. , & Mullins, C. D. (2017). Framework for enhancing clinical practice guidelines through continuous patient engagement. Health Expectations, 20, 3–10. 27115476 10.1111/hex.12467 PMC5217879

[cl21444-bib-0005] Arnstein, S. R. (1969). A ladder of citizen participation. Journal of the American Institute of Planners, 35(4), 216–224.

[cl21444-bib-0006] Boivin, A. , Currie, K. , Fervers, B. , Gracia, J. , James, M. , Marshall, C. , Sakala, C. , Sanger, S. , Strid, J. , Thomas, V. , van der Weijden, T. , Grol, R. , & Burgers, J. (2010). Patient and public involvement in clinical guidelines: International experiences and future perspectives. BMJ Quality & Safety, 19(5), e22. 10.1136/qshc.2009.034835 20427302

[cl21444-bib-0007] Boivin, A. , L'Espérance, A. , Gauvin, F. P. , Dumez, V. , Macaulay, A. C. , Lehoux, P. , & Abelson, J. (2018). Patient and public engagement in research and health system decision making: A systematic review of evaluation tools. Health Expectations, 21(6), 1075–1084. 30062858 10.1111/hex.12804 PMC6250878

[cl21444-bib-0008] van de Bovenkamp, H. M. , & Zuiderent‐Jerak, T. (2015). An empirical study of patient participation in guideline development: Exploring the potential for articulating patient knowledge in evidence‐based epistemic settings. Health Expectations, 18(5), 942–955. 23634973 10.1111/hex.12067 PMC5060867

[cl21444-bib-0009] Brett, J. , Staniszewska, S. , Mockford, C. , Herron‐Marx, S. , Hughes, J. , Tysall, C. , & Suleman, R. (2014a). A systematic review of the impact of patient and public involvement on service users, researchers and communities. The Patient – Patient‐Centred Outcomes Research, 7(4), 387–395. 10.1007/s40271-014-0065-0 25034612

[cl21444-bib-0010] Brett, J. , Staniszewska, S. , Mockford, C. , Herron‐Marx, S. , Hughes, J. , Tysall, C. , & Suleman, R. (2014b). Mapping the impact of patient and public involvement on health and social care research: A systematic review. Health Expectations, 17(5), 637–650. 22809132 10.1111/j.1369-7625.2012.00795.x PMC5060910

[cl21444-bib-0011] Brouwers, M. C. , Kho, M. E. , Browman, G. P. , Burgers, J. S. , Cluzeau, F. , Feder, G. , Fervers, B. , Graham, I. D. , Grimshaw, J. , Hanna, S. E. , Littlejohns, P. , Makarski, J. , & Zitzelsberger, L. (2010). AGREE II: Advancing guideline development, reporting and evaluation in health care. Canadian Medical Association Journal, 182(18), E839–E842. 20603348 10.1503/cmaj.090449 PMC3001530

[cl21444-bib-0012] Campbell, B. , Tabiri‐Essuman, J. , Gallo, H. , Verdiel, V. , Mandava, L. , Azhar, M. A. , & Powell, J. (2017). Public consultation changes guidance on the use of health‐care interventions. An observational study. Health Expectations, 20(2), 361–368. 27312870 10.1111/hex.12476 PMC5354025

[cl21444-bib-0013] Campbell, M. , McKenzie, J. E. , Sowden, A. , Katikireddi, S. V. , Brennan, S. E. , Ellis, S. , Hartmann‐Boyce, J. , Ryan, R. , Shepperd, S. , Thomas, J. , Welch, V. , & Thomson, H. (2020). Synthesis without meta‐analysis (SWiM) in systematic reviews: Reporting guideline. BMJ, 368, l6890. 31948937 10.1136/bmj.l6890 PMC7190266

[cl21444-bib-0014] Cluzeau, F. , Wedzicha, J. A. , Kelson, M. , Corn, J. , Kunz, R. , Walsh, J. , & Schünemann, H. J. (2012). Stakeholder involvement: How to do it right: Article 9 in integrating and coordinating efforts in COPD guideline development. An official ATS/ERS workshop report. Proceedings of the American Thoracic Society, 9(5), 269–273. 23256170 10.1513/pats.201208-062ST

[cl21444-bib-0015] Concannon, T. W. , Grant, S. , Welch, V. , Petkovic, J. , Selby, J. , Crowe, S. , Synnot, A. , Greer‐Smith, R. , Mayo‐Wilson, E. , Tambor, E. , & Tugwell, P. (2019). Practical guidance for involving stakeholders in health research. Journal of General Internal Medicine, 34(3), 458–463. 30565151 10.1007/s11606-018-4738-6 PMC6420667

[cl21444-bib-0016] Concannon, T. W. , Meissner, P. , Grunbaum, J. A. , McElwee, N. , Guise, J.‐M. , Santa, J. , Conway, P. H. , Daudelin, D. , Morrato, E. H. , & Leslie, L. K. (2012). A new taxonomy for stakeholder engagement in patient‐centered outcomes research. Journal of General Internal Medicine, 27(8), 985–991. 22528615 10.1007/s11606-012-2037-1 PMC3403141

[cl21444-bib-0017] Crocker, J. C. , Boylan, A. M. , Bostock, J. , & Locock, L. (2017). Is it worth it? Patient and public views on the impact of their involvement in health research and its assessment: A UK‐based qualitative interview study. Health Expectations, 20(3), 519–528. 27338242 10.1111/hex.12479 PMC5433537

[cl21444-bib-0018] Crowe, S. (2017). Who inspired my thinking?—Sherry Arnstein. Research for All, 1(1), 143–146.

[cl21444-bib-0019] Deeks, J. J. , Higgins, J. P. T. , & Altman, D. G. (Eds.). (2023). Cochrane Handbook for Systematic Reviews of Interventions version 6.4 (updated August 2023). Cochrane.

[cl21444-bib-0020] Del Campo, P. D. , Gracia, J. , Blasco, J. , & Andradas, E. (2011). A strategy for patient involvement in clinical practice guidelines: Methodological approaches. BMJ Quality & Safety, 20(9), 779–784. 10.1136/bmjqs.2010.049031 21460393

[cl21444-bib-0021] Dukhanin, V. , Topazian, R. , & DeCamp, M. (2018). Metrics and evaluation tools for patient engagement in healthcare organization‐and system‐level decision‐making: A systematic review. International Journal of Health Policy and Management, 7(10), 889–903. 30316241 10.15171/ijhpm.2018.43 PMC6186472

[cl21444-bib-0022] Flemming, K. , Booth, A. , Garside, R. , Tunçalp, Ö. , & Noyes, J. (2019). Qualitative evidence synthesis for complex interventions and guideline development: Clarification of the purpose, designs and relevant methods. BMJ Global Health, 4(Suppl. 1), e000882. 10.1136/bmjgh-2018-000882 PMC6350756 30775015

[cl21444-bib-0023] Forsythe, L. , Heckert, A. , Margolis, M. K. , Schrandt, S. , & Frank, L. (2018). Methods and impact of engagement in research, from theory to practice and back again: Early findings from the Patient‐Centered Outcomes Research Institute. Quality of Life Research, 27(1), 17–31. 28500572 10.1007/s11136-017-1581-x PMC5770504

[cl21444-bib-0024] Fraenkel, L. , Miller, A. S. , Clayton, K. , Crow‐Hercher, R. , Hazel, S. , Johnson, B. , Rott, L. , White, W. , Wiedmeyer, C. , Montori, V. M. , Singh, J. A. , & Nowell, W. B. (2016). When patients write the guidelines: Patient panel recommendations for the treatment of rheumatoid arthritis. Arthritis Care & Research, 68(1), 26–35. 10.1002/acr.22758 26545701 PMC4715535

[cl21444-bib-0025] Google LLC . (2024). Google Sheets. Retrieved from https://sheets.google.com

[cl21444-bib-0026] Greenhalgh, T. , Hinton, L. , Finlay, T. , Macfarlane, A. , Fahy, N. , Clyde, B. , & Chant, A. (2017). Frameworks for supporting patient and public involvement in research: Systematic review and co‐design pilot. Health Expectations, 3(1), 19. 10.1111/hex.12888 PMC6737756 31012259

[cl21444-bib-0027] Gupta, M. , McCauley, J. , Farkas, A. , Gudeloglu, A. , Neuberger, M. M. , Ho, Y.‐Y. , Yeung, L. , Vieweg, J. , & Dahm, P. (2015). Clinical practice guidelines on prostate cancer: A critical appraisal. Journal of Urology, 193(4), 1153–1158. 25451831 10.1016/j.juro.2014.10.105

[cl21444-bib-0028] Guyatt, G. H. , Oxman, A. D. , Vist, G. E. , Kunz, R. , Falck‐Ytter, Y. , Alonso‐Coello, P. , & Schünemann, H. J. (2008). GRADE: An emerging consensus on rating quality of evidence and strength of recommendations. BMJ, 336(7650), 924–926. 18436948 10.1136/bmj.39489.470347.AD PMC2335261

[cl21444-bib-0029] Haddaway, N. R. , Grainger, M. J. , & Gray, C. T. (2022). Citationchaser: A tool for transparent and efficient forward and backward citation chasing in systematic searching. Research Synthesis Methods, 13(4), 533–545. 35472127 10.1002/jrsm.1563

[cl21444-bib-0030] Hamilton, C. B. , Hoens, A. M. , McKinnon, A. M. , McQuitty, S. , English, K. , Hawke, L. D. , & Li, L. C. (2021). Shortening and validation of the Patient Engagement In Research Scale (PEIRS) for measuring meaningful patient and family caregiver engagement. Health Expectations, 24(3), 863–879. 33729634 10.1111/hex.13227 PMC8235891

[cl21444-bib-0031] Hamilton, C. B. , Hoens, A. M. , McQuitty, S. , McKinnon, A. M. , English, K. , Backman, C. L. , Azimi, T. , Khodarahmi, N. , & Li, L. C. (2018). Development and pre‐testing of the Patient Engagement In Research Scale (PEIRS) to assess the quality of engagement from a patient perspective. PLoS One, 13(11), e0206588. 30383823 10.1371/journal.pone.0206588 PMC6211727

[cl21444-bib-0032] Harrison, R. , Jones, B. , Gardner, P. , & Lawton, R. (2021). Quality assessment with diverse studies (QuADS): An appraisal tool for methodological and reporting quality in systematic reviews of mixed‐ or multi‐method studies. BMC Health Services Research, 21(1), 144. 10.1186/s12913-021-06122-y 33588842 PMC7885606

[cl21444-bib-0033] Hoekstra, F. , Mrklas, K. J. , Khan, M. , McKay, R. C. , Vis‐Dunbar, M. , Sibley, K. M. , Nguyen, T. , Graham, I. D. , & Gainforth, H. L. (2020). A review of reviews on principles, strategies, outcomes and impacts of research partnerships approaches: A first step in synthesising the research partnership literature. Health Research Policy and Systems, 18(1), 51. 32450919 10.1186/s12961-020-0544-9 PMC7249434

[cl21444-bib-0034] Khabsa, J. , Petkovic, J. , Riddle, A. , Lytvyn, L. , Magwood, O. , Atwere, P. , Campbell, P. , Katikireddi, S. V. , Merner, B. , Nasser, M. , Chang, S. , Jaramillo Garcia, A. , Limburg, H. , Guise, J. M. , Tugwell, P. , & Akl, E. A. (2022). PROTOCOL: Conflict of interest issues when engaging stakeholders in health and healthcare guideline development: A systematic review. Campbell Systematic Reviews, 18(2), e1232. 36911340 10.1002/cl2.1232 PMC9013401

[cl21444-bib-0035] Kunz, R. , Fretheim, A. , Cluzeau, F. , Wilt, T. J. , Qaseem, A. , Lelgemann, M. , Kelson, M. , Guyatt, G. , & Schünemann, H. J. (2012). Guideline group composition and group processes: Article 3 in integrating and coordinating efforts in COPD guideline development. An official ATS/ERS workshop report. Proceedings of the American Thoracic Society, 9(5), 229–233. 23256164 10.1513/pats.201208-056ST

[cl21444-bib-0036] Légaré, F. , Boivin, A. , van der Weijden, T. , Pakenham, C. , Burgers, J. , Légaré, J. , St‐Jacques, S. , & Gagnon, S. (2011). Patient and public involvement in clinical practice guidelines: A knowledge synthesis of existing programs. Medical Decision Making, 31(6), E45–E74. 21959267 10.1177/0272989X11424401

[cl21444-bib-0037] Lewin, S. , Booth, A. , Glenton, C. , Munthe‐Kaas, H. , Rashidian, A. , Wainwright, M. , Bohren, M. A. , Tunçalp, Ö. , Colvin, C. J. , Garside, R. , Carlsen, B. , Langlois, E. V. , & Noyes, J. (2018). Applying GRADE‐CERQual to qualitative evidence synthesis findings: Introduction to the series. Implementation Science, 13(1), 2. 10.1186/s13012-017-0688-3 29384079 PMC5791040

[cl21444-bib-0038] Magwood, O. , Riddle, A. , Petkovic, J. , Lytvyn, L. , Khabsa, J. , Atwere, P. , Akl, E. A. , Campbell, P. , Welch, V. , Smith, M. , Mustafa, R. A. , Limburg, H. , Dans, L. F. , Skoetz, N. , Grant, S. , Concannon, T. W. , & Tugwell, P. (2022). Barriers and facilitators to stakeholder engagement in health guideline development: A qualitative evidence synthesis. Campbell Systematic Reviews, 18(2), e1237. 36911345 10.1002/cl2.1237 PMC9038083

[cl21444-bib-0039] Masterson, D. , Areskoug Josefsson, K. , Robert, G. , Nylander, E. , & Kjellström, S. (2022). Mapping definitions of co‐production and co‐design in health and social care: A systematic scoping review providing lessons for the future. Health Expectations, 25, 902–913. 35322510 10.1111/hex.13470 PMC9122425

[cl21444-bib-0040] Minogue, V. , Cooke, M. , Donskoy, A.‐L. , Vicary, P. , & Wells, B. (2018). Patient and public involvement in reducing health and care research waste. Research Involvement and Engagement, 4(1), 5. 29449962 10.1186/s40900-018-0087-1 PMC5808395

[cl21444-bib-0041] Mockford, C. , Staniszewska, S. , Griffiths, F. , & Herron‐Marx, S. (2012). The impact of patient and public involvement on UK NHS health care: A systematic review. International Journal for Quality in Health Care, 24(1), 28–38. 22109631 10.1093/intqhc/mzr066

[cl21444-bib-0042] Mrklas, K. J. , Boyd, J. M. , Shergill, S. , Merali, S. , Khan, M. , Nowell, L. , Goertzen, A. , Pfadenhauer, L. M. , Paul, K. , Sibley, K. M. , Swain, L. , Vis‐Dunbar, M. , Hill, M. D. , Raffin‐Bouchal, S. , Tonelli, M. , & Graham, I. D. (2023). Tools for assessing health research partnership outcomes and impacts: A systematic review. Health Research Policy and Systems, 21(1), 3. 36604697 10.1186/s12961-022-00937-9 PMC9817421

[cl21444-bib-0043] Mrklas, K. J. , Merali, S. , Khan, M. , Shergill, S. , Boyd, J. M. , Nowell, L. , Pfadenhauer, L. M. , Paul, K. , Goertzen, A. , Swain, L. , Sibley, K. M. , Vis‐Dunbar, M. , Hill, M. D. , Raffin‐Bouchal, S. , Tonelli, M. , & Graham, I. D. (2022). How are health research partnerships assessed? A systematic review of outcomes, impacts, terminology and the use of theories, models and frameworks. Health Research Policy and Systems, 20(1), 133. 36517852 10.1186/s12961-022-00938-8 PMC9753311

[cl21444-bib-0044] Neumann, I. , Santesso, N. , Akl, E. A. , Rind, D. M. , Vandvik, P. O. , Alonso‐Coello, P. , Agoritsas, T. , Mustafa, R. A. , Alexander, P. E. , Schünemann, H. , & Guyatt, G. H. (2016). A guide for health professionals to interpret and use recommendations in guidelines developed with the GRADE approach. Journal of Clinical Epidemiology, 72, 45–55. 26772609 10.1016/j.jclinepi.2015.11.017

[cl21444-bib-0045] Oliver, S. R. , Rees, R. W. , Clarke‐Jones, L. , Milne, R. , Oakley, A. R. , Gabbay, J. , Stein, K. , Buchanan, P. , & Gyte, G. (2008). A multidimensional conceptual framework for analysing public involvement in health services research. Health Expectations, 11(1), 72–84. 18275404 10.1111/j.1369-7625.2007.00476.x PMC5060424

[cl21444-bib-0046] O'Neill, J. , Tabish, H. , Welch, V. , Petticrew, M. , Pottie, K. , Clarke, M. , Evans, T. , Pardo Pardo, J. , Waters, E. , White, H. , & Tugwell, P. (2014). Applying an equity lens to interventions: Using PROGRESS ensures consideration of socially stratifying factors to illuminate inequities in health. Journal of Clinical Epidemiology, 67(1), 56–64. 24189091 10.1016/j.jclinepi.2013.08.005

[cl21444-bib-0047] Page, M. J. , McKenzie, J. E. , Bossuyt, P. M. , Boutron, I. , Hoffmann, T. C. , Mulrow, C. D. , Shamseer, L. , Tetzlaff, J. M. , Akl, E. A. , Brennan, S. E. , Chou, R. , Glanville, J. , Grimshaw, J. M. , Hróbjartsson, A. , Lalu, M. M. , Li, T. , Loder, E. W. , Mayo‐Wilson, E. , McDonald, S. , … Moher, D. (2021). The PRISMA 2020 statement: An updated guideline for reporting systematic reviews. BMJ, 372(n), n71. 33782057 10.1136/bmj.n71 PMC8005924

[cl21444-bib-0048] Petkovic, J. , Riddle, A. , Akl, E. A. , Khabsa, J. , Lytvyn, L. , Atwere, P. , Campbell, P. , Chalkidou, K. , Chang, S. M. , Crowe, S. , Dans, L. , Jardali, F. E. , Ghersi, D. , Graham, I. D. , Grant, S. , Greer‐Smith, R. , Guise, J. M. , Hazlewood, G. , Jull, J. , … Tugwell, P. (2020). Protocol for the development of guidance for stakeholder engagement in health and healthcare guideline development and implementation. Systematic Reviews, 9(1), 21. 32007104 10.1186/s13643-020-1272-5 PMC6995157

[cl21444-bib-0049] Petkovic, J. , Riddle, A. , Lytvyn, L. , Khabsa, J. , Akl, E. A. , Welch, V. , Magwood, O. , Atwere, P. , Graham, I. D. , Grant, S. , John, D. , Katikireddi, S. V. , Langlois, E. , Mustafa, R. A. , Todhunter‐Brown, A. , Schünemann, H. , Smith, M. , Stein, A. T. , Concannon, T. , & Tugwell, P. (2022). PROTOCOL: Guidance for stakeholder engagement in guideline development: A scoping review. Campbell Systematic Reviews, 18(2), e1242. 36911343 10.1002/cl2.1242 PMC9096120

[cl21444-bib-0050] Pollock, A. , Campbell, P. , Struthers, C. , Synnot, A. , Nunn, J. , Hill, S. , Goodare, H. , Morris, J. , Watts, C. , & Morley, R. (2019). Development of the ACTIVE framework to describe stakeholder involvement in systematic reviews. Journal of Health Services Research & Policy, 24(4), 245–255. 30997859 10.1177/1355819619841647

[cl21444-bib-0051] Schünemann, H. J. , Wiercioch, W. , Etxeandia, I. , Falavigna, M. , Santesso, N. , Mustafa, R. , Ventresca, M. , Brignardello‐Petersen, R. , Laisaar, K. T. , Kowalski, S. , Baldeh, T. , Zhang, Y. , Raid, U. , Neumann, I. , Norris, S. L. , Thornton, J. , Harbour, R. , Treweek, S. , Guyatt, G. , … Akl, E. A. (2014). Guidelines 2.0: Systematic development of a comprehensive checklist for a successful guideline enterprise. Canadian Medical Association Journal, 186(3), E123–E142. 24344144 10.1503/cmaj.131237 PMC3928232

[cl21444-bib-0052] Stallings, S. C. , Boyer, A. P. , Joosten, Y. A. , Novak, L. L. , Richmond, A. , Vaughn, Y. C. , & Wilkins, C. H. (2019). A taxonomy of impacts on clinical and translational research from community stakeholder engagement. Health Expectations, 22(4), 731–742. 31321849 10.1111/hex.12937 PMC6737764

[cl21444-bib-0053] Staniszewska, S. , Adebajo, A. , Barber, R. , Beresford, P. , Brady, L. M. , Brett, J. , Elliott, J. , Evans, D. , Haywood, K. L. , Jones, D. , Mockford, C. , Nettle, M. , Rose, D. , & Williamson, T. (2011). Developing the evidence base of patient and public involvement in health and social care research: The case for measuring impact. International Journal of Consumer Studies, 35(6), 628–632.

[cl21444-bib-0054] Staniszewska, S. , Brett, J. , Simera, I. , Seers, K. , Mockford, C. , Goodlad, S. , Altman, D. G. , Moher, D. , Barber, R. , Denegri, S. , Entwistle, A. , Littlejohns, P. , Morris, C. , Suleman, R. , Thomas, V. , & Tysall, C. (2017). GRIPP2 reporting checklists: Tools to improve reporting of patient and public involvement in research. Research Involvement and Engagement, 3(1), 13. 29062538 10.1186/s40900-017-0062-2 PMC5611595

[cl21444-bib-0055] Steinberg, E. , Greenfield, S. , Wolman, D. M. , Mancher, M. , & Graham, R. (2011). Clinical practice guidelines we can trust. National Academies Trust. 24983061

[cl21444-bib-0056] Tong, A. , Flemming, K. , McInnes, E. , Oliver, S. , & Craig, J. (2012). Enhancing transparency in reporting the synthesis of qualitative research: ENTREQ. BMC Medical Research Methodology, 12(1), 181. 10.1186/1471-2288-12-181 23185978 PMC3552766

[cl21444-bib-0057] Vat, L. E. , Finlay, T. , Robinson, P. , Barbareschi, G. , Boudes, M. , Diaz Ponce, A. M. , Dinboeck, M. , Eichmann, L. , Ferrer, E. , Fruytier, S. E. , Hey, C. , Broerse, J. E. W. , & Schuitmaker‐Warnaar, T. J. (2021). Evaluation of patient engagement in medicine development: A multi‐stakeholder framework with metrics. Health Expectations, 24(2), 491–506. 33629470 10.1111/hex.13191 PMC8077089

[cl21444-bib-0058] World Health Organization . *WHO Handbook for guideline development*. World Health Organization.

